# MaxSSmap: a GPU program for mapping divergent short reads to genomes with the maximum scoring subsequence

**DOI:** 10.1186/1471-2164-15-969

**Published:** 2014-11-15

**Authors:** Turki Turki, Usman Roshan

**Affiliations:** Computer Science Department, King Abdulaziz University, P.O. Box 80221, Jeddah, Saudi Arabia; Department of Computer Science, New Jersey Institute of Technology, GITC 4400 University Heights, Newark USA

**Keywords:** Alignment, Divergent, GPU, NGS

## Abstract

**Background:**

Programs based on hash tables and Burrows-Wheeler are very fast for mapping short reads to genomes but have low accuracy in the presence of mismatches and gaps. Such reads can be aligned accurately with the Smith-Waterman algorithm but it can take hours and days to map millions of reads even for bacteria genomes.

**Results:**

We introduce a GPU program called MaxSSmap with the aim of achieving comparable accuracy to Smith-Waterman but with faster runtimes. Similar to most programs MaxSSmap identifies a local region of the genome followed by exact alignment. Instead of using hash tables or Burrows-Wheeler in the first part, MaxSSmap calculates maximum scoring subsequence score between the read and disjoint fragments of the genome in parallel on a GPU and selects the highest scoring fragment for exact alignment. We evaluate MaxSSmap’s accuracy and runtime when mapping simulated Illumina *E.coli* and human chromosome one reads of different lengths and 10% to 30% mismatches with gaps to the *E.coli* genome and human chromosome one. We also demonstrate applications on real data by mapping ancient horse DNA reads to modern genomes and unmapped paired reads from NA12878 in 1000 genomes.

**Conclusions:**

We show that MaxSSmap attains comparable high accuracy and low error to fast Smith-Waterman programs yet has much lower runtimes. We show that MaxSSmap can map reads rejected by BWA and NextGenMap with high accuracy and low error much faster than if Smith-Waterman were used. On short read lengths of 36 and 51 both MaxSSmap and Smith-Waterman have lower accuracy compared to at higher lengths. On real data MaxSSmap produces many alignments with high score and mapping quality that are not given by NextGenMap and BWA. The MaxSSmap source code in CUDA and OpenCL is freely available from http://www.cs.njit.edu/usman/MaxSSmap.

**Electronic supplementary material:**

The online version of this article (doi:10.1186/1471-2164-15-969) contains supplementary material, which is available to authorized users.

## Background

In next generation sequencing experiments we may encounter divergent reads in various scenarios. These include structural variation studies, comparison of distantly related genomes, absence of same species reference genome, sequence error in long reads, genome variation within same species, ancient DNA mapping, and mRNA-seq experiments [1–10]. Programs [11, 12] based on hash tables and Burrows-Wheeler transform are very fast but have low accuracy on such reads that tend to contain many mismatches and gaps [1, 13]. The Smith-Waterman algorithm [14] can map divergent reads accurately but is considerably expensive. Even high performance multi-core and Graphics Processing Unit (GPU) implementations can take hours and days to align millions of reads even to bacteria genomes. As a solution we introduce a GPU program called MaxSSmap with the aim of achieving comparable accuracy to Smith-Waterman on divergent reads but with faster runtimes.

We divide the genome into same size disjoint fragments and then map a read to all fragments in parallel on a GPU with the maximum scoring subsequence score [15, 16]. A GPU can run several hundred threads at the same time and allows for massive parallelism in computer programs (see http://www.gpucomputing.net). The maximum scoring subsequence is roughly the same as Smith-Waterman except that it does not consider gaps.

Once we identify the first and second highest scoring fragments — we need the second to eliminate repeats — we perform Needleman-Wunsch alignment of the read to the identified region of genome. We present a GPU program called MaxSSmap that implements this idea along with several heuristics and shortcuts that lead to faster runtimes without sacrificing accuracy.

On reads with fewer than 10% mismatches our program offers no advantage over hash-table approaches. Programs like NextGenMap that use hash-tables in their first phase can map such reads very quickly with high accuracy compared to other leading programs [13]. Thus we focus on reads with divergence between 10% and 30% as well as gaps of lengths up to 30 both in the read and genome.

We compare MaxSSmap to two fast Smith-Waterman programs. The first is the recently published Smith-Waterman library for short read mapping and protein database search called SSW [17]. This uses a fast Single-Instruction-Multiple-Data Smith-Waterman algorithm to align a given read to the entire genome. The authors of the program demonstrate improved and comparable runtimes to state of the art fast Smith-Waterman programs for mapping DNA sequences to a genome. In addition this produces output in SAM format and has also been applied it to real data in the context of realigning unmapped reads [17]. The second is a fast GPU Smith-Waterman program for protein sequence database search called CUDA-SW++ [18]. We note that this is not designed for mapping DNA sequence reads. However, we adapt it to short read mapping by considering fragments of the genome as database sequences and read as the query.

Exact Smith-Waterman methods take much longer than hash-table and Burrows-Wheeler based programs to align millions of reads to genome sequences. In this setting we study several meta-methods that first align reads with a fast program and then map rejected ones with a slower but more accurate one such as MaxSSmap and SSW.

We study accuracy and runtime for mapping simulated Illumina *E.coli* and human reads of various lengths to the *E.coli* and human chromosome one. Our focus is on reads with 10% to 30% mismatches and gaps up to length 30. We show that MaxSSmap attains comparable high accuracy and low error as CUDA-SW++ and SSW but is several fold faster than the two programs respectively. We show that MaxSSmap can map reads rejected by NextGenMap [13] with high accuracy and low error and much faster than if Smith-Waterman were used. We also study MaxSSmap on various read lengths and demonstrate applications on real data by mapping ancient horse DNA reads to modern genomes and unpaired mapped reads in 1000 genomes subject NA12878.

Below we provide basic background and describe our program in detail. We then present our experimental results on simulated and real data.

## Implementation

We provide implementations of our program for CUDA versions 4.2 and 6.0 and for OpenCL version 1.1. We use a freely available open source library called SimpleOpenCL (from https://code.google.com/p/simple-opencl/ that provides an OpenCL interface that siginficantly reduces the amout of host code. We use the libOpenCL.so library from CUDA 6.5 release for running the OpenCL implementation on NVIDIA GPUs. Our OpenCL implementation has a Makefile to compile on AMD and Intel GPUs as well.

## Methods

### Background

Before we describe MaxSSmap we provide background on the maximum scoring subsequence and GPUs, CUDA, and OpenCL.

#### Maximum scoring subsequence

The maximum scoring subsequence for a sequence of real numbers {*x*_1_,*x*_2_,…,*x*_*n*_} is defined to be the contiguous subsequence {*x*_*i*_,…,*x*_*j*_} that maximizes the sum *x*_*i*_+…+*x*_*j*_ (0≤*i*,*j*≤*n*). A simple linear time approach will find the maximum scoring subsequence [15, 16]. To apply this to DNA sequences consider two of the same length aligned to each other without gaps. Each aligned character corresponds to a substitution whose cost can be obtained from a position specific scoring matrix that accounts for base call probabilities, or a substitution scoring matrix, or a trivial match or mismatch cost. The maximum scoring subsequence between the two DNA sequences can now be obtained through this sequence of substitution scores [15, 16].

#### Graphics processing units (GPUs)

The GPU is designed for running in parallel hundreds of short functions called threads. Threads are organized into blocks which in turn are organized into grids. We use one grid and automatically set the number of blocks to the total number of genome fragments divided by the number of threads to run in a block. The number of threads in a block can be specified by the user and otherwise we set it to 256 by default.

The GPU memory is of several types each with different size and access times: global, local, constant, shared, and texture. Global memory is the largest and can be as much as 6GB for Tesla GPUs. Local memory is the same as global memory but limited to a thread. Access times for global and local memory are much higher than the those for a CPU program to access RAM. However, this time can be considerably reduced with coalescent memory access that we explain below. Constant and texture are cached global memory and accessible by any thread in the program. Shared is on-chip making it the fastest and is limited to threads in a block.

More details about the GPU architecture can be found in the NVIDIA online documentation [19] and recent books [20, 21].

#### CUDA and OpenCL

CUDA is a programming language that is developed by NVIDIA. It is mainly C with extensions for programming only on NVIDIA GPUs. OpenCL [22] is a framework for writing computer programs that execute on different platforms that include GPUs and CPUs.

### MaxSSmap algorithm

#### Overview

Our program, that we call MaxSSmap, follows the same two part approach of existing mappers: first identify a local region of the genome and then align the read with Needleman-Wunsch (or Smith-Waterman) to the identified region. The second part is the same as current methods but in the first part we use the maximum scoring subsequence as described below.

MaxSSmap divides the genome into fragments of a fixed size given by the user. It uses one grid and automatically sets the number of blocks to the total number of genome fragments divided by the number of threads to run in a block. The number of threads in a block can be specified by the user and otherwise we set it to 256 by default.

##### First phase of MaxSSmap

**Figure 1:**
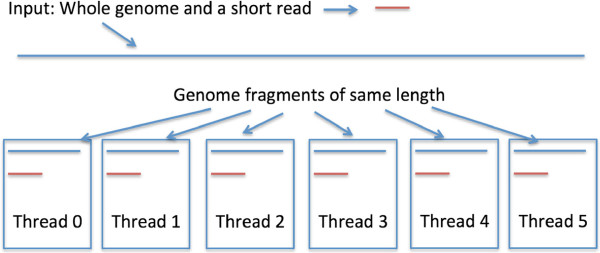
**Overview of the MaxSSmap program.** In this figure the genome is divided into six fragments which means six threads will run on the GPU. Thread with ID 0 maps the read to fragment 0, slides it across fragment 0, and stops when it has covered all of fragment 0. We account for junctions between fragments and ensure that the read is fully mapped to the genome.

In Figure 1 we show an overview of the MaxSSmap program. Each thread of the GPU computes the maximum scoring subsequence [15] of the read and a unique fragment with a sliding window approach. In order to map across junctions between fragments each thread also considers neighboring fragments when mapping the read. When done it outputs the fragment number with the highest and second highest score and considers the read to be mapped if the ratio of the second best to best score is below.9 (chosen by empirical performance). This reduces false positives due to repeats. We later define a mapping quality score that is based on this ratio.

##### Second phase of MaxSSmap

After the fragment number is identified we consider the region of the genome starting from the identified fragment and spanning fragments to the right until we have enough nucleotides as the read sequence. In the second part we align the read sequence with Needleman-Wunsch to the genome region from the first part. The default settings for match, mismatch, and gap costs that we also use in this study are set to 5, -4, and -26.

##### Incorporating base qualities and position specific scoring matrix

We also consider the base qualities of reads in both phases of the program. This can be done easily by creating a position specific scoring matrix for each read that also allows for fast access using table lookup [18]. For example let *x* be the probability that the base at position *i* is correctly sequenced. This can be calculated by the phred score [23] that is provided with the reads. The score of a match against the nucleotide at position *i* is *match*×*x* and mismatch is .

##### Mapping qualities, read lengths, SAM output, and source code

MaxSSmap outputs in SAM format that is widely used for mapping DNA reads to genomes [24]. In the MAPQ field of SAM [24] we use the formula −100 log2*p* where *p* is the probability of alignment being incorrect. We define this to be the ratio of the scores of the second highest and top scoring fragments. For MaxSSmap we consider the read mapped only if mapping quality is above −100 log2.9=15.2. MaxSSmap can also map reads of various lengths present in one fastq file. There is no need to specify the read length. However, the maximum read length is limited to 2432 base pairs (bp) in the current implementation (see paragraph on shared memory below). The source code is freely available from http://www.cs.njit.edu/usman/MaxSSmap.

We implement several novel heuristics and take advantage of the GPU architecture to speed up our program which we describe below.

#### GPU specific heuristics

##### Coalescent global memory access

**Figure 2:**
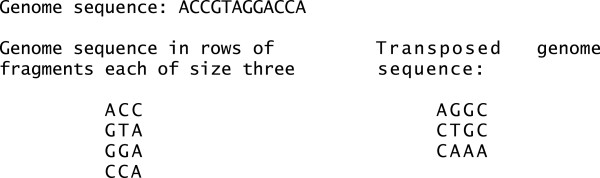
**Genome sequence in transpose format to enable coalescent memory access.** In MaxSSmap threads with IDs 0 through 3 would at the same time read characters A, G, G, and C of the transposed genome to compare against the read. Since the four characters are in consecutive memory locations and so are the thread IDs, our program makes just one read from global memory instead of four separate ones.

Coalesced memory access is a key performance consideration when programming on GPUs (see the CUDA C Best Practices Guide [25]). Roughly speaking, each thread of a GPU has its own unique identifier that we call *thread*_*id*. In order to have coalescent memory access our program must have threads with consecutive identifiers access consecutive locations in memory (roughly speaking). We achieve this by first considering the genome sequence as rows of fragments of a fixed size. We then transpose this matrix to yield a transposed genome sequence that allows coalescent memory access. The transposed genome is transferred just once in the beginning of the program from CPU RAM to GPU global memory. It has negligible overhead time compared to the total one for mapping thousands of reads. See Figure 2 for a toy genome ACCGTAGGACCA and fragment length of three. If the genome is not a multiple of the fragment length we pad the last fragment with N’s. Our GPU program runs a total of *numfragments* threads. In the example shown in Figure 2 there are four fragments. And so our program would run four threads simultaneously with identifiers zero through three. Each thread would access the transposed genome sequence first at location *thread*_*id*, then at *thread*_*id*+*numfragments*, followed by location *thread*_*id*+2*numfragments*, and so on.

##### Byte packing for faster global memory access

In the GPU we store the genome sequence in a single array of int4 type instead of char. This leads to fewer global memory accesses and thus faster runtimes. To enable this we append ‘N’ characters onto the genome and query until both lengths are multiples of 16. This also requires that the fragment length be a multiple of 16.

##### Look ahead strategy to reduce global memory penalties

As mentioned earlier MaxSSmap uses a sliding window approach from left to right to map a read to a given fragment on the genome. In its implementation we compute the score of the read in the current window and sixteen windows to the right at the same time. Therefore instead of shifting the window by one nucleotide we shift it by sixteen. This leads to fewer global memory calls and also allows us to unroll loops. See file MaxSSMap_shared_int4_fast.cu in the source code for exact implementation.

##### Shared memory

We store the query in shared memory to allow fast access. As mentioned earlier the GPU access time to shared memory is fastest. This, however, imposes a limitation on the read length because shared memory size is much smaller than global memory. The Fermi Tesla M2050 GPUs that we use in this study have a maximum of 49152 bytes shared memory per block. The data structure stores the query in a profile format and so occupies a total of (*readlength*+16)×4×5 bytes. The 4 accounts for number of bytes in a float, 5 is for bases A, C, G, T, and N, and 16 is for additional space used by the look-ahead strategy and to eliminate if-statements in the code. Thus the maximum allowable DNA read length of the current implementation is 2432 bp (largest multiple of 16 below the cap size of 2441 bp). The query length can be increased at the expense of running time by storing the query in constant memory, which is of size 65536 byes, or in global memory.

#### Parallel multi-threaded CPU implementation of MaxSSmap

We have also implemented a parallel multi-threaded CPU implementation of MaxSSmap with the OpenMP library [26] (OpenMP available from http://www.openmp.org). Each thread maps the given read to a unique fragment of the genome. The number of threads is automatically set to the genome size divided by the specified fragment length. Thus if the fragment length is 4800 then for *E.coli* (approximately 5 million bp) it runs about 1042 threads on the available CPU cores. This also uses the look ahead strategy as described above. However, the coalescent and shared memory techniques don’t apply to this version since they are specific to a GPU.

### Programs compared and their versions and parameters

The literature contains many short read alignment programs that have been benchmarked extensively [11, 12]. Instead of considering many different programs we select the widely used program BWA [27] that uses the Burrows-Wheeler transform. We also select NextGenMap that uses hash-tables and is shown to be accurate on reads upto 10% mismatches compared to other leading programs [13]. We use the multi-threaded version of BWA and enable the GPU option in NextGenMap.

Other GPU programs for mapping short reads [28–31] are implementations of CPU counterparts designed for speedup and achieve the same accuracy. Since they offer no improvement in accuracy they would perform poorly on divergent reads. Furthermore, the CPU program runtimes are already in seconds vs. minutes and hours for exact methods (such as ours and Smith-Waterman) and so we exclude these programs from the paper.

From the category of exact mapping programs we use SSW [17] that uses a fast Single-Instruction-Multiple-Data (SIMD) Smith-Waterman algorithm to align a given read to the entire genome and the fast GPU Smith-Waterman program CUDA-SW++ [18]. As noted earlier this is designed for protein sequence database search and not for aligning to large genome sequences. However, we adapt it to short read mapping by considering fragments of the genome as database sequences and read as the query.

Below we describe program parameters and how we optimized them where applicable. The exact command line of each program is given in the Online Additional file 1 at http://www.cs.njit.edu/usman/MaxSSmap.

#### MaxSSmap

For MaxSSmap we consider fragment lengths of 48 for *E.coli* genome, 480 for human chromosome one, 4800 for horse and whole human genomes, and match and mismatch costs of 5 and -4 respectively. In the exact alignment phase where we perform Needleman-Wunsch we consider the same match and mismatch cost and a gap cost of -26. We selected fragment lengths to optimize runtime. We considered sizes of 16, 32, 48, 64, and 80 for the *E.coli* genome, lengths of 160, 240, 320, 400, and 480 for human chromosome one, and lengths of 2400, 3600, 4800, 6000, and 7200 for the horse genome. For the whole human genome we used the same fragment size as for the horse genome. The match and mismatch costs are optimized for accuracy on the 251bp length *E.coli* reads. For other genomes we recommend the user to experiment with different fragment sizes starting with a small value. As explained earlier the MaxSSmap fragment length must be a multiple of 16 because of byte packing to allow storage of the genome in an array of int4 instead of char.

#### MaxSSmap_fast

In this faster version of MaxSSmap we consider every other nucleotides in the read sequence when mapping to the genome. This heuristic reduces runtime considerably than if we were to compare all nucleotides in the read sequence.

See files MaxSSMap_shared_int4_fast.cu in the source code for exact implementation.

#### SSW

This is a recent Smith-Waterman library that uses Single-Instruction-Multiple-Data (SIMD) to achieve parallelism. It has been shown to be faster than other SIMD based Smith-Waterman approaches [17]. It has also been applied to real data as a secondary program to align reads rejected by primary programs [17].

#### CUDA-SW++

CUDA-SW++ [18] is originally designed for protein database search. It performs Smith-Waterman alignment of the query to each sequence in the database in parallel. We simulate short read mapping with it by dividing the genome into same size disjoint fragments and considering each fragment of the genome as one of the database sequences and the read as the query. We set CUDA-SW++ to output the two top highest scoring fragments and their scores. If the ratio of the second best score to the best one is above.9 we do not consider the read mapped. We set the fragment length to 512 and 2400 for the *E.coli* genome and horse genomes, the gap open and extension costs to -26 and -1, and the match and mismatch costs to 5 and -4. These values yielded highest accuracy for the simulated reads. We modified the code so that the blosum45 matrix uses +5 for match and -4 for mismatch. We choose 512 fragment length for *E.coli* because lower ones reduce the runtime marginally but the accuracy goes down considerably whereas higher fragment lengths don’t yield higher accuracy and increase runtime. The gap, match, and mismatch costs are optimized for accuracy on the 251bp *E.coli* reads. For the horse genome we couldn’t run CUDA-SW++ with higher fragment lengths of 4800 and so we selected 2400.

#### BWA-MEM

We use BWA-MEM version 0.7.5a with multi-threaded enabled (-t 12) and other options set to their default values.

#### NextGeneMap

We use NextGeneMap version 0.4.10 with the options -g 0 that enables the GPU and everything else default.

#### Meta-methods

We consider four meta-methods that first apply a NextGenMap and then a more accurate aligner for rejected reads.

- NextGenMap + MaxSSmap
- NextGenMap + MaxSSmap_fast
- NextGenMap + CUDASW++
- NextGenMap + SSW

We use the same options for each program in the meta-method as described above.

### Experimental platform

All programs were executed on Intel Xeon X5650 machines with 12GB RAM each equipped with three NVIDIA Tesla M2050 GPUs with 3GB global memory and 49152 byes of shared memory. We used CUDA release 4.2 to develop MaxSSmap and to compile and build the GPU programs. For our OpenCL implementation we use version 1.1. In Table 1 we list the architecture on which we run each program.

**Table 1:** **Architecture for each program compared in our study**

Program	Architecture
MaxSSmap	GPU
MaxSSmap_fast	GPU
CUDA-SW++	GPU
SSW	SIMD single CPU (GPU unavailable)
NextGenMap	GPU
BWA-MEM	Multi-threaded 12 CPUs

### Data simulation

We use the program Stampy [1] (version 1.0.22) to simulate reads with realistic base qualities. We use the *E.coli* genome K12 MG1665 (4.6 million bp) from which Stampy simulates reads and Illumina MiSeq 251bp reads in SRR522163 from the NCBI Sequence Read Archive (http://www.ncbi.nlm.nih.gov/Traces/sra) from which Stampy simulates base qualities. For the human reads we use the human chromosome one sequence from the Genome Reference Consortium (http://www.ncbi.nlm.nih.gov/projects/genome/assembly/grc/) version GRCh37.p13. We use Illumina MiSeq 250bp reads in ERR315985 through ERR315997 from the the NCBI Sequence Read Archive from which Stampy simulated base qualities.

We simulate one million 251 bp *E.coli* reads and 250bp human chromosome one reads of divergences 0.1, 0.2, and 0.3 with and without gaps ranging upto length 30. The gaps are randomly chosen to occur in the read or the genome. Roughly speaking each divergence corresponds to fraction of mismatches in the reads after accounting for sequencing error. For example.1 divergence means on average 10% mismatches excluding sequencing errors. See Table 2 for exact Stampy command line parameters for simulating the data.

**Table 2:** **Stampy (version 1.0.22) parameters to simulate reads**

Genome	Stampy parameters
*E.coli* (format genome)	-G ecoli ecoli_K12_MG1665.fasta
(hash)	-g ecoli -H ecoli
(simulate)	-g ecoli -h ecoli -S SRR522163_1.fastq
Human (format genome)	-G hs_ref_GRCh37.p13_chr1
	hs_ref_GRCh37.p13_chr1.fa
(hash)	-g hs_ref_GRCh37.p13_chr1
	-H hs_ref_GRCh37.p13_chr1
(simulate)	-g hs_ref_GRCh37.p13_chr1
	-h hs_ref_GRCh37.p13_chr1
	-S ERR315985_to_ERR315997_1.fastq
Divergence	
.1	–substitutionrate=.1
.2	–substitutionrate=.2
.3	–substitutionrate=.3
.1+gaps	–substitutionrate=.1 –simulate-minindellen=-30
	–simulate-maxindellen=30 –insertsize=250
	–insertsd=25
.2+gaps	–substitutionrate=.2 –simulate-minindellen=-30
	–simulate-maxindellen=30 –insertsize=250
	–insertsd=25
.3+gaps	–substitutionrate=.3 –simulate-minindellen=-30
	–simulate-maxindellen=30 –insertsize=250
	–insertsd=25

### Measure of accuracy and error

For Stampy simulated reads the true alignment is given in a CIGAR string format [24]. Except for CUDA-SW++ we evaluate the accuracy of all programs with the same method used in [1]. We consider the read to be aligned correctly if at least one of the nucleotides in the read is aligned to the same one in the genome as given by the true alignment. It’s not unusual to allow a small window of error as done in previous studies (see [12] for a thorough discussion).

CUDA-SW++ does not output in SAM format. Instead it gives the top scoring fragments and the score of the query against the fragment. To evaluate its accuracy we divide the true position by the fragment size which is 512 for *E.coli* and 2400 for horse genome in our experiments. We then consider the read to be mapped correctly if the difference between the CUDA-SW++ fragment and the true one is at most 1.

## Results

We study the accuracy and runtime of all programs and the four meta-methods described earlier. In all experiments below we use the CUDA 4.2 executable of MaxSSmap when comparing it against other programs. We include a subsection that specifically compares the CUDA 4.2, CUDA 6.0, and OpenCL implementations of MaxSSmap. We measure their performance for mapping simulated Illumina *E.coli* and human reads to the *E.coli* and human chromosome one respectively. We then compare them on reads of different lengths and demonstrate applications on real data.

### Comparison of MaxSSmap and Smith-Waterman for mapping divergent reads to *E.coli*genome

We begin by comparing MaxSSmap and MaxSSmap_fast to SSW and CUDA-SW++. We map 100,000 251bp simulated *E.coli* reads to the *E.coli* genome. We simulate these reads using the Stampy [1] program (described earlier).

As mentioned earlier, MaxSSmap offers no advantage over hash-table approaches on reads with fewer than 10% mismatches. Programs like NextGenMap [13] designed for mapping to polymorphic genomes can align such reads very quickly with high accuracy. Thus we consider three levels of divergence in the reads: 0.1, 0.2, and 0.3. Roughly speaking each divergence corresponds to the percentage of mismatches in the data.

In Table 3(a) we see that the MaxSSmap accuracy is comparable to SSW and CUDA-SW++ except at divergence 0.3 with gaps (our hardest setting). Table 3(b) shows that the MaxSSmap and MaxSSmap_fast runtimes are at least 44 and 60 times lower than SSW and 5.8 and 8 times lower than CUDA-SW++. This is where the real advantage of MaxSSmap lies: high accuracy and low error comparable to Smith-Waterman on reads up to 30% mismatches and gaps yet at a lower cost of runtime.

**Table 3:** **Comparison of MaxSSmap and MaxSSmap_fast to a GPU and a SIMD high performance Smith-Waterman implementation**

(a) Percent of 100,000 251 bp reads mapped correctly to the ***E.coli*** genome. Shown in parenthesis are incorrectly mapped reads and remaining are rejected				
**Div**	**MaxSSmap_fast**	**MaxSSmap**	**CUDASW++**	**SSW**
**Reads without gaps**				
.1	95 (0.4)	96 (0.4)	94 (0.9)	97 (3)
.2	95 (0.6)	95.3 (0.6)	94 (1)	97 (3)
.3	90 (1.1)	94.2 (0.9)	93 (1.3)	96 (4)
**Reads with gaps**				
.1	92 (1.5)	93.1 (1.9)	94 (0.9)	97 (3)
.2	90 (1.7)	92.5 (2.1)	92 (1)	96 (4)
.3	81 (2.8)	89.9 (3.5)	92 (1.4)	95 (5)
**(b) Time in minutes to map 100,000 251 bp reads to the** ***E.coli*** **genome**				
**Div**	**MaxSSmap_fast**	**MaxSSmap**	**CUDASW++**	**SSW**
**Reads without gaps**				
.1	20	28	164	1288
.2	20	28	164	1275
.3	20	28	164	1255
**Reads with gaps**				
.1	20	28	163	1283
.2	20	28	162	1266
.3	20	28	162	1235

These are simulated Illumina reads and contain realistic base qualities generated from Illumina short reads. Each divergence represents the average percent of mismatches in the reads. So 0.1 means 10% mismatches on the average. The gaps are randomly chosen to occur in the read or the genome and are of length at most 30.

At high divergence and with gaps we expect Smith-Waterman to fare better in accuracy and error than our maximum scoring subsequence heuristic. For example at divergence 0.3 with gaps SSW is 5.1% and 14% better than MaxSSmap and MaxSSmap_fast in accuracy.

Recall that MaxSSmap detects and rejects repeats which are likely to be errors. We use the same technique in the CUDA-SW++ output. However, SSW does not appear to have such a strategy and so we see a higher error for it.

### Comparison of meta-methods for mapping divergent *E.coli*reads

We now compare the accuracy and runtime of four meta-methods that use NextGenMap in the first phase of mapping and MaxSSmap, MaxSSmap_fast, CUDASW++, and SSW to align rejected reads in the second phase. We study the mapping of one million 251 bp reads simulated *E.coli* reads to the *E.coli* genome.

In Table 4 we see that the accuracy of NGM+CUDASW++ and NGM+SSW are comparable to NGM+MaxSSmap but runtimes are much higher. For example at divergence 0.2 with gaps NGM+SSW takes over 48 hours to finish and NGM+CUDASW++ takes 756 minutes, whereas NGM+MaxSSmap and NGM+MaxSSmap_fast finish in 109 and 68 minutes respectively. At divergence 0.3 with gaps both NGM+MaxSSmap and NGM+MaxSSmap_fast finish within four hours whereas both NGM+CUDASW++ and NGM+SSW take more than 24 hours. We choose the two fastest meta-methods for comparison to BWA and NextGenMap.

**Table 4:** **Comparison of meta-methods**

(a) Percent of one million 251 bp reads mapped correctly to the***E.coli*** genome. Shown in parenthesis are incorrectly mapped				
reads and remaining are rejected				
**Div**	**NextGenMap+**	**NextGenMap+**	**NextGenMap+**	**NextGenMap+**
	**MaxSSmap_fast**	**MaxSSmap**	**CUDASW++**	**SSW**
		**Reads without gaps**		
.1	96 (1.3)	97 (1.4)	97 (1)	97 (2.8)
.2	95 (1.5)	96.7 (1.5)	96 (1)	NA
.3	91 (1.4)	94.8 (1.3)	93 (1.5)	NA
		**Reads with gaps**		
.1	92 (1.5)	95.7 (1.4)	97 (1)	97.2 (2.8)
.2	92 (2.1)	94 (2.5)	95 (2)	NA
.3	82 (2.9)	90.5 (3.5)	92.5 (1.6)	NA
**(b) Time in minutes to map one million 251 bp reads to the** ***E.coli*** **genome**				
**Div**	**NextGenMap+**	**NextGenMap+**	**NextGenMap+**	**NextGenMap+**
	**MaxSSmap_fast**	**MaxSSmap**	**CUDASW++**	**SSW**
		**Reads without gaps**		
.1	25	34	197	1397
.2	57	77	537	NA
.3	148	204	1343	NA
		**Reads with gaps**		
.1	38	60	413	2601
.2	68	109	756	NA
.3	162	222	1528	NA

See Table 3 caption for details about reads. NA denotes time greater than 48 hours which is our cutoff time on this data.

### Comparison of fastest meta-methods to NextGenMap and BWA for mapping divergent *E.coli*and human chromosome one reads

In Tables 5 and 6 we compare the accuracy and runtimes of NextGenMap and BWA to NGM+MaxSSmap and NGM+MaxSSmap_fast. Both meta-methods achieve high accuracy and low error at all settings but at the cost of increased runtime compared to NextGenMap and BWA. On *E.coli* reads of divergence 0.1 and 0.2 with gaps NextGenMap+MaxSSmap_fast yields an improvement of 14% and 32% over NextGenMap while adding 37 and 66 minutes to the NextGenMap time of 1.5 and 2 minutes respectively. On human chromosome 1 reads of the same settings NextGenMap+MaxSSmap_fast correctly aligns an additional 2% and 13% reads than NextGenMap alone at the cost of 3.2 and 582 extra minutes. The MaxSSmap runtimes for human chromosome 1 are higher than for *E.coli* because there are many more fragments to consider in the former. The runtimes for both meta-methods also increases with higher divergence because there are many more reads rejected by NextGenMap at those divergences.

**Table 5:** **Comparison of meta-methods to NextGenMap and BWA**

(a) Percent of one million 251 bp reads mapped correctly to the***E.coli*** genome. Shown in parenthesis are incorrectly mapped reads and remaining are rejected				
**Div**	**BWA**	**NextGenMap**	**NextGenMap+**	**NextGenMap+**
			**MaxSSmap_fast**	**MaxSSmap**
	**Reads without gaps**			
.1	89 (1.1)	87 (1)	96 (1.3)	97 (1.4)
.2	24 (0.5)	72 (1)	95 (1.5)	96.7 (1.5)
.3	0.6 (0)	26 (0.5)	91 (1.4)	94.8 (1.3)
	**Reads with gaps**			
.1	85 (3)	78 (1)	92 (1.5)	95.7 (1.4)
.2	20 (0.5)	60 (1)	92 (2.1)	94 (2.5)
.3	0.5 (0)	19 (0.4)	82 (2.9)	90.5 (3.5)
**(b) Time in minutes to map one million 251 bp reads to the** ***E.coli*** **genome**				
**Div**	**BWA**	**NextGenMap**	**NextGenMap+**	**NextGenMap+**
			**MaxSSmap_fast**	**MaxSSmap**
	**Reads without gaps**			
.1	0.7	1.2	25	34
.2	0.5	1.9	57	77
.3	0.4	2.1	148	204
	**Reads with gaps**			
.1	0.7	1.5	38	60
.2	0.5	2.1	68	109
.3	0.4	2.1	162	222

See Table 3 caption for details about reads.

**Table 6:** **Comparison of meta-methods to NextGenMap and BWA**

(a) Percent of one million 250 bp reads mapped correctly to the human chromosome one genome. Shown in parenthesis are incorrectly mapped reads and remaining are rejected			
**Div**	**BWA**	**NextGenMap**	**NextGenMap+MaxSSmap_fast**
**Reads without gaps**			
.1	96(3)	99 (1)	99 (1)
.2	33 (6)	86 (6)	94 (6.2)
.3	0.8 (6)	37 (9)	88 (10.5)
**Reads with gaps**			
.1	89 (3)	96 (2)	98 (2)
.2	28 (5)	79 (6)	92 (6.5)
.3	0.7 (0.5)	30 (7)	80 (9.4)
**(b) Time in minutes to map one million 250 bp reads to the human chromosome one genome**			
**Div**	**BWA**	**NextGenMap**	**NextGenMap+MaxSSmap_fast**
**Reads without gaps**			
.1	1.6	7.1	10.3
.2	0.8	44	626
.3	0.6	65	3992
**Reads with gaps**			
.1	1.5	9.8	176
.2	0.7	51	1126
.3	0.6	66	4611

See Table 3 caption for details about reads.

NextGenMap has higher accuracy than BWA as shown here in Tables 5 and 6 and in previous studies [13] while BWA is the fastest program amongst all compared. We ran BWA in a multi-threaded mode that utilizes all CPU cores and all other methods on the GPU. We found that running NextGenMap on the GPU was faster than its multi-threaded mode.

### Comparison of methods on reads of various lengths

The simulated sequences in our above results are based upon Illumina MiSeq sequences of lengths 250 and 251 bp. Here we study simulated reads of lengths 36, 51, 76, 100, and 150 based on real E.coli sequences from the Illumina Genome Analyzer II (36 bp), HiSeq 1000 (51 bp), HiSeq 2000 (76 and 100 bp), and MiSeq (150 bp). We obtained the sequences from datasets ERR019652 (36 bp), SRR1016504 (51 bp), SRR1016920 (76 bp), ERR376625 (100 bp), and SRR826444 and SRR826446 (150 bp) in the NCBI Sequence Read Archive (http://www.ncbi.nlm.nih.gov/Traces/sra). We simulated 1 million reads of each length from the *E.coli* genome and of divergence 0.1 with gaps (up to length 30) with Stampy as described earlier. Recall that Stampy simulated base qualities based upon the real data in theinput.

In Table 7 we compare the accuracy and runtimes of BWA, NextGenMap, NextGenMap+MaxSSmap_fast, and NextGenMap+MaxSSmap. As the read length increases we see that NextGenMap and the meta-methods increase in accuracy and decrease in error. BWA is the most conservative and has lowest error at all lengths especially on the shortest read lengths.

**Table 7:** **Percent of one million reads of lengths 36, 51, 76, 100, and 150 and of divergence 0.1 with gaps mapped correctly to the** ***E.coli*** **genome**

Read	BWA	NGM	NGM+	NGM+	NGM+
length			MaxSSmap_fast	MaxSSmap	CUDA-SW++
36	1.2 (0)	33.3 (10.5)	34 (16.5)	43.8 (18.1)	53.2 (17.1)
51	11.4 (0.2)	50.5 (3)	53.3 (8.8)	71.2 (7.5)	79.5 (6.2)
76	38.4 (.7)	70.2 (1.5)	76.5 (4.3)	92.9 (2.7)	96.6 (1.8)
100	62.4 (1)	82.6 (1.4)	91 (2.2)	96.8 (1.9)	98.1 (1.5)
150	76 (1.1)	82.5 (1.2)	92 (2.2)	93.8 (2.2)	94.7 (2.1)
**Time in minutes to map reads**					
36	0.09	0.3	26.6	37.8	315.9
51	0.11	0.5	26.6	38.2	284.9
76	0.15	0.6	19.8	28.4	201.9
100	0.22	0.7	14.8	21	129.9
150	0.34	0.9	19.6	29.2	173.8

Shown in parenthesis are incorrectly mapped reads and remaining are rejected. We denote NextGenMap by NGM.

At reads lengths of 100 and above NextGenMap+MaxSSmap has about 10% higher accuracy than NextGenMap and 1% more error.

### Comparison to parallel multi-threaded CPU implementation of MaxSSmap

We also study the runtimes of the parallel multi-threaded CPU implementation of MaxSSmap as described earlier. We examined three fragments lengths of 4800, 48000, and 480000. Each yields 1042, 104, and 11 threads to run on available CPU cores. We ran this program on Intel Xeon CPU which has a total of 12 cores.

We tested this for mapping a 100,000 251 bp *E.coli* reads and found fragment length of 4800 to be the fastest. We then mapped 100,000 251 bp *E.coli* reads which took 224 minutes. In comparison the GPU MaxSSmap takes 20 minutes. Thus we find the multi-threaded version to be 10 times slower.

### Applications on real data

We consider two scenarios in real data where unmapped divergent reads may occur. The first is in the mapping of ancient fossil DNA to modern genomes and second is the alignment of unmapped reads when comparing a human genome to the standard reference.

#### Ancient horse DNA mapping to modern genomes

For the first case we consider reads obtained from an ancient horse bone [32]. In a previous study the parameters of the BWA program were optimized to maximize mapped reads from this set to the horse and human genomes [33]. We consider one set of reads from the same study in dataset SRR111892 obtained from the NIH Sequence Read Archive (http://www.ncbi.nlm.nih.gov/Traces/sra). These reads are produced by the Illumina Genome Analyzer II sequencer and have an average length of 67.7 and standard deviation of 8.4 We obtained the human genome from the Genome Reference Consortium (http://www.ncbi.nlm.nih.gov/projects/genome/assembly/grc/) version GRCh37.p13 and the horse genome Equus_caballus EquCab2 (GCA_000002305.1) from Ensemble (http://useast.ensembl.org/Equus_caballus/Info/Index).

Highly divergent sequences are likely to be present in this dataset and as we have seen in the previous section short read lengths of up to 76 are challenging even with 10% mismatches and gaps. Thus we consider only reads of the maximum length of 76 in this dataset. We map the first 100000 such reads with BWA, NextGenMap, NextGenMap+MaxSSmap_fast, NextGenMap+MaxSSmap, and NextGenMap+CUDASW++ to the horse and human genomes. We consider the human genomes to identify ancient human DNA fragments in the sample [9].

In Table 8 we see that NextGenMap aligns 16% and 14% of the reads to the horse and human genomes whereas NextGenMap+MaxSSmap aligns a total of 23.1% and 21% respectively. BWA in comparison aligns 2.2% and 0.16% with default parameters and has similar accuracy with optimized parameters given in a previous study [33].

**Table 8:** **Percent of 100,000 ancient horse DNA reads (SRR111892) of length 76 bp mapped to the horse genome Equus_caballus EquCab2 (GCA_000002305.1) and human reference genome**

Horse genome				
**BWA**	**NextGenMap**	**NextGenMap+**	**NextGenMap+**	**NextGenMap+**
		**MaxSSmap_fast**	**MaxSSmap**	**CUDASW++**
2.2	16	20.5	23.1	26 (estimated)
		**Time in minutes to map reads**		
0.6	2.4	1609.6	2836	14820 (estimated)
**Human genome**				
**BWA**	**NextGenMap**	**NextGenMap+**	**NextGenMap+**	**NextGenMap+**
		**MaxSSmap_fast**	**MaxSSmap**	**CUDASW++**
0.16	14	18.6	21	NA
		**Time in minutes to map reads**		
0.82	2.9	2375.5	4108	NA

We ran NextGenMap+CUDA-SW++ for a maximum of 168 hours and estimated the time to align all rejected reads. Also shown is time in minutes.

We evaluated NextGenMap+CUDA-SW++ for mapping reads to the horse genome by running it for a maximum of 168 hours (one week). In that time period CUDA-SW++ considered 56800 of the 84036 NextGenMap rejected reads to be aligned (about 68%) and mapped 11.8% of them. Based on this we estimate it would take 247 hours for CUDA-SW++ to consider all of the NextGenMap rejected reads. And it would align a total of 11.8% of 84036 reads which equals 9916. This added to the NextGenMap mapped reads gives a total mapping rate of 26%.

To better understand these numbers we simulated 100,000 horse 76 bp reads of divergence 40% and gaps with Stampy and with base qualities simulated from the same real dataset (SRR111892) used here. These settings were chosen to achieve a ballpark mapping rate with the real data. We find that NextGenMap aligns 18% of total reads with only 0.4% correctly mapped while BWA maps no reads at all. These are similar to the mapping rates on the real data. This suggests these are difficult settings for NextGenMap and BWA. When we apply MaxSSmap to reads missed by NextGenMap it aligns an additional 8.8% (similar to the real data) with 3.3% correct. To reduce the error to zero we raise the MaxSSmap mapping quality threshold from the default 15.2 to 62. Raising it gives fewer but higher quality mapped reads. This gives 0.112% mapped reads all of which are correct.

With this in mind we return to the real data and apply NextGenMap+MaxSSmap with the higher mapping quality cutoff (of 62) in the hopes of obtaining all correct alignments. The higher threshold gives 9 additional reads. In the Additional file 1 we give the SAM output of these alignments. Most have at least 30% mismatches and challenging for both BWA and NextGenMap.

#### Mapping unaligned reads from NA12878 to human reference

For our second scenario we consider the popular human genome sequence NA12878 and study the mapping of one of its dataset SRR16607 to the same human reference used above. We map the first 100000 paired reads in SRR016607 (101 bp) with NextGenMap, NextGenMap+MaxSSmap_fast, and NextGenMap+MaxSSmap (fragment length set to 4800). In the latter two methods we re-align the pairs with MaxSSmap_fast and MaxSSmap where at least one read in the pair was unmapped by NextGenMap or the mapped pair positions were outside the mapping distance threshold of 500 base pairs.

In Table 9 we measure the number of paired reads whose mapped positions are within 500 base pairs. Although the nominal insert size of this dataset is 300 with a standard deviation of 77 (as given in the NIH SRA website) we found many mapped pairs in the output that were within 500 base pairs and so we use this threshold. These mapped pairs are called concordant reads [1]. We see that NextGenMap aligns 83.5% of the reads concordantly whereas NextGenMap+MaxSSmap_fast and NextGenMap+MaxSSmap align 85.8% and 87.6%. Both methods mapped 0.7% and 1.2% pairs discordantly (mapped positions at least 500 bp apart). NextGenMap mapped 9.4% of the pairs discordantly but we don’t report this here because we re-align those reads with MaxSSmap.

**Table 9:** **Percent of 100,000 paired human reads from NA12878 in 1000 genomes (SRR016607) of length 101 bp mapped concordantly to the human genome**

NextGenMap	NextGenMap+	NextGenMap+
	MaxSSmap_fast	MaxSSmap
83.5 (0)	85.8 (0.7)	87.6 (1.2)
**Time in minutes to map paired reads**		
1.5	1295.9	2242.4

Concordant reads are pairs that are mapped within 500 base pairs. Also shown in parenthesis are discordant reads (mapped positions at least 500 bp apart) and the time in minutes. In NextGenMap+MaxSSmap we re-align pairs with MaxSSmap where at least one read in the pair was unmapped by NextGenMap or the pair is discordant. Thus, NextGenMap shows zero discordant pairs because we re-align them with MaxSSmap.

### Comparison of CUDA and OpenCL executables of MaxSSmap

We compare the CUDA and OpenCL MaxSSmap exectuables for mapping reads to *E.coli*, horse, and human genomes. Both implementations produce similar output and the same accuracies and error rates given earlier. In Table 10 we see that the CUDA 6.0 runtimes are lowest followed by OpenCL and CUDA 4.2. The differences are small when mapping to E.coli but larger for the horse and human genomes.

**Table 10:** **Running time comparison of CUDA and OpenCL implementations of MaxSSmap (denoted by MSS)**

(a) Time in minutes to map 100,000 251 bp reads to the ***E.coli*** genome. See Table 3 caption for details about reads						
**Div**	**MSS_fast**	**MSS**	**MSS_fast**	**MSS**	**MSS_fast**	**MSS**
**CUDA 4.2**			**CUDA 6.0**		**OpenCL**	
**Reads without gaps**						
.1	20	28	17	27	17	27
.2	20	28	17	27	17	27
.3	20	28	17	27	17	27
**Reads with gaps**						
.1	20	28	17	27	17	27
.2	20	28	17	27	17	27
.3	20	28	17	27	17	27
**(b) Time in minutes to map paired human reads from NA12878 in 1000 genomes (SRR016607) of length 101 bp to the human**						
**genome. We denote NextGenMap by NGM and MaxSSmap by MSS. See Table** 8 **for more details about reads**						
**NGM+**	**NGM+**	**NGM+**	**NGM+**	**NGM+**	**NGM+**	
**MSS_fast**	**MSSmap**	**MSS_fast**	**MSS**	**MSS_fast**	**MSS**	
**CUDA 4.2**		s		**OpenCL**		
1295.9	2242.4	1183.5	2092.7	1252.7	2159.9	
**(c) Time in minutes to map 100,000 ancient horse DNA reads (SRR111892) of length 76 bp to**						
**the horse genome Equus_caballus EquCab2 (GCA_000002305.1). See Table** 9 **for more details**						
**about reads**						
**NGM+**	**NGM+**	**NGM+**	**NGM+**	**NGM+**	**NGM+**	
**MSS_fast**	**MSSmap**	**MSS_fast**	**MSS**	**MSS_fast**	**MSS**	
**CUDA 4.2**		**CUDA 6.0**		**OpenCL**		
1609.6	2836	1515	2689.8	1561.8	2736.8	

The output from the three methods give the same accuracies and errors as given earlier but the running times vary. We find the CUDA 6.0 implementation to have the lowest runtimes followed by OpenCL and CUDA 4.2.

## Discussion

In our experimental results we have demonstrated the advantage of MaxSSmap over Smith-Waterman for mapping reads to genomes. In scenarios where accurate re-alignment of rejected and low-scoring reads are required MaxSSmap and MaxSSmap_fast would be fast alternatives to Smith-Waterman. Such conditions are likely to contain reads with many mismatches and gaps which would get rejected by programs based on hash-tables and Burrows-Wheeler.

We demonstrate two such scenarios on real data. In both cases we see an increase in mapped reads by MaxSSmap. While this increase comes at the cost of considerable runtime it is still much faster than the Smith-Waterman alternative. Furthermore, the output of NextGenMap+MaxSSmap reveals many high scoring alignments well above the mapping quality threshold that warrant further study.

The MaxSSmap alignments of horse DNA reads to the horse and human genomes contain 39.3% and 39.4% mismatches on the average. In the human genome paired read study we find the MaxSSmap concordant aligned pairs to contain 19.1% mismatches on the average. In both cases these are challenging divergences for BWA and NextGenMap as we saw in the simulation studies.

Our real data applications in this paper are brief and deserve a wider study in a separate paper. For example when we aligned unmapped pairs in NA12878 to the human genome we obtained 4.1% more pairs with MaxSSmap. We will search for variants in these alignments as part of future work. Also in future work we plan to study MaxSSmap on metagenomic reads where divergence rates can be high as well.

Our results on both real and simulated data are an insight into missed reads that are rejected by BWA and NextGenMap. We see that the high mismatch rate and gaps are the main reasons why these reads are unmapped in the first place. Without more exact approaches like MaxSSmap and Smith-Waterman it would be much harder to align such reads.

The ratio of the NextGenMap+CUDASW++ to the NextGenMap+MaxSSmap runtimes varies and can depend upon number of reads to align. In Table 4 where reads lengths are fixed at 251 bp we see that this ratio is 5.8 at divergence 0.1 without gaps where both MaxSSmap and CUDA-SW++ align approximately 12,000 reads (that number rejected by NextGenMap in Table 5). As the divergence increase to 0.3 with gaps both programs align about 800,000 reads and there the ratio of their runtimes is 6.9.

Our program is not without limitations. We find that at very short reads of lengths 36 and 51 the improvements given by MaxSSmap are small compared to higher lengths.

However, read lengths of 125 and above are not uncommon especially since current Illumina machines such as MiSeq, HiSeq, and NextSeq generate reads of at least this length (see http://www.illumina.com/systems/sequencing.ilmn)

We find the runtimes are much higher for human and horse chromosomes than for *E.coli* just because there are many more genome fragments for the former. This could be lowered by spreading reads across multiple GPUs. We also see that the accuracy of MaxSSmap is lower than Smith-Waterman as we cross into higher divergence of 0.3 with gaps.

## Conclusion

We introduce a GPU program called MaxSSmap for mapping reads to genomes. We use the maximum scoring subsequence to identify candidate genome fragments for final alignment instead of hash-tables and Burrows-Wheeler transform. We show that MaxSSmap has comparable high accuracy to Smith-Waterman based programs yet has lower runtimes and accurately maps reads rejected by a hash-table based mapper faster than if Smith-Waterman were used. We also study MaxSSmap on different read lengths and demonstrate applications on real data by mapping ancient horse DNA reads to modern genomes and unmapped paired reads from NA12878 in 1000 genomes.

## Availability and requirements

**Project name:** MaxSSmap**Project home page:**http://www.cs.njit.edu/usman/MaxSSmap**Operating system(s):** Linux (tested on Red Hat Enterprise Linux 6.2)**Programming language:** CUDA versions 4.2 and 6.0 and OpenCL version 1.1**Other requirements:** CUDA toolkits version 4.2 or 6.0, GNU gcc C compiler (tested on version 4.4.7) to compile OpenCL executable**License:** The MIT License

## Electronic supplementary material

Additional file 1: **Program command lines and high quality ancient horse DNA aligned reads given by MaxSSmap.** (PDF 68 KB)
