# Supplementary material for: MaxSSmap: a GPU program for mapping divergent short reads to genomes with the maximum scoring subsequence
Source: BMC Genomics. 2014 Nov 15;15(1):969. doi: 10.1186/1471-2164-15-969 (PMC4289234; doi:10.1186/1471-2164-15-969)
Supplement: Supplementary file 1 — Additional file 1: Program command lines and high quality ancient horse DNA aligned reads given by MaxSSmap. (PDF 68 KB) [file 12864_2014_6854_MOESM1_ESM.pdf]

Supplementary Material for ”MaxSSmap: A GPU program for mapping divergent short reads to genomes with the maximum scoring subsequence”

Turki Turki[*] Usman Roshan[†‡]

Program command lines for mapping reads

MaxSSmap ./MaxSSMap dnagenome -q <reads in FASTQ format> -f ecoli_K12_MG1665.fasta -cd 0 -nq 100000 -t 256 -fl 48. For human chromsome one genome mapping we set -fl to 480 instead of 48.

SSW ./ssw_test -o 26 -e 1 -m 5 -x 4 -s -c -r ecoli_K12_MG1665.fasta <reads in FASTQ format>

CUDA-SW++ ./cudasw -mat blosum45 -query <input reads in FASTA format and with reverse complement> -db ecoli_K12_MG1665.fasta.fixed_512fragments -gapo 26 -gape 1 -topscore_num 2 -min_score 10 -use_single 0

BWA-MEM bwa/bwa mem data/ecoli_K12_MG1665.fasta <reads in FASTQ format> -t 12

NextGeneMap ngm -q <reads in FASTQ format> -r
ecoli_K12_MG1665.fasta -g 0

High quality ancient horse DNA reads aligned by MaxSSmap

```
@SRR111892.21029 16 >1 74744332 68.241013 80M0I * 0 0 NNNNGCTGAACCGCTCTTCCGATCTCGCCGACGATGTTCTTGCCGATGTCATGCACGTCGCCCTTCACCGTGGCGATGAG
NNNN=>A??<@A@@AAEA>AAAABAEEBEEEEEEEHEEEFHBHEHFHFEHHHHHHHHHHGHHFGGGFGBGGGGEEE-GGGGG AS:128 MATCHES:48 MISMATCHES:27 GAPS:1

@SRR111892.70499 0 >10 28374023 65.021797 80M0I * 0 0 TCGACGACCTGATCACCCTGGGTACCCAGGAACCGTACCGGATGTTCACGATCGGAAGAGCGGTTCAGCAGGAATGNNNNN
HHBGHHBHHHHHDFFFGGGGGGGGGHHHHHBBGGGGHHGHHFGHH428(224977HHDHEFHFEHFGCDGEDGFEENNNN AS:141 MATCHES:50 MISMATCHES:26 GAPS:0

@SRR111892.79451 0 >8 65158345 64.831848 53M1I7M1I18M0I * 0 0 CACGGCCTGGTGGACGGCGCAGCAGCAGGGAAATCTGGCGGTAAGCCAGATCGGAAGAGCGGTTCAGCAGGAATGCNNNN
HHHHHGDGGG=AC>?EEEFEGGGGGC8<CEGGEBGEHHHHF>GFFAA<C>BBBBBECB8CDB><@>>;>@>DBBB>NNNN AS:134 MATCHES:54 MISMATCHES:20 GAPS:2
```

[*] Computer Science Department, King Abdulaziz University, P.O. Box 80221, Jeddah 21589, Saudi Arabia
[†] To whom correspondence should be addressed; usman@cs.njit.edu, Ph: 862-485-0590, Fax: 973-596-5777
[‡] Department of Computer Science, New Jersey Institute of Technology, Newark, NJ 07102

```
@SRR111892.107301 0 >14 36248342 94.446465 80M0I * 0 0 CCTTATTGAGCAGCGCGAAATCCAACCCCTTGACCAAGTGCGTGTGGGCCAGGTCTCCGCCCAAGTACTTGGACGCNNNN
IIIIIIIIIIIIIIIIHIIIIIIIIIIIHHIIIGIIIIIIIIIEIHIIIIHIHDHHEIHIIGIIHFDDBHEFEEDGDEFNNNN AS:205 MATCHES:57 MISMATCHES:19 GAPS:0

@SRR111892.132623 0 >23 49116114 84.803749 80M0I * 0 0 CCGAAGGTTTCGAACGCATTCACCGCACCAACCTGGTGGGCATGGGCGTGTTGCCGCTGGAGTTCCTGCCGGGCACNNNN
IIHIIIIIIIIIIIIIIHIGIIIIIIIIIIIIIHBGGGGIIIHFIHGGIHIIGIIHIIIIIHIIEHEIGGIGFEDBEGHGNNNN AS:187 MATCHES:55 MISMATCHES:21 GAPS:0

@SRR111892.139309 0 >11 26139901 63.845387 80M0I * 0 0 TCGCGGTGGCGAGAACGTGTACCCGCGCGAGTTGGAAGAGTTTTTCTTCACGCACCCGGCGGTGGCCGATGTGCAGNNNN
IIIIHIIIHHIIIIIIIIIIFIIIIIHIIIIFEGGGGFIIDIFIIIIHIIFHIGHIHIGGEIHDDEEEDEDDDA>BEENNNN AS:178 MATCHES:54 MISMATCHES:22 GAPS:0
@SRR111892.302467 0 >5 32460075 68.194588 80M0I * 0 0 TCATTGTCGCCTTGAAAGCGTGGCGTCATCATCTGATGGGCTCGAAGAGTTCGGAAGAGCGGTTCAGCAGGAATGCNNNN
:EFBBDBBDD<FFFBB4EB@GGGGGG4G@>BB<FCBG3GDFGGB3::8:+8682+=:>/0*0011C>>A9>CC>>DNNNN AS:160 MATCHES:52 MISMATCHES:24 GAPS:0

@SRR111892.323666 0 >15 19870215 63.447102 80M0I * 0 0 CTGGCAAGCGCATGGGCCATGCTGGCGCAATCATCTCTGGCGGCAAAGGCACTGCAGACGAGAAATTCGCTGCACTNNNN
IIIIIIIIIIIIIIIIIFIIIIIIHIIIIIIIIIHHIHIIIIHIIIGHIIHFEHHIEEHIGGHIIGIHHGGEDEEDGGDGNNNN AS:169 MATCHES:53 MISMATCHES:23 GAPS:0

@SRR111892.333217 0 >2 43475636 63.941010 80M0I * 0 0 ACAAGGCTGGAGATTCGTGTCCTCTGGGCGTGGACTGAGATCGGAAGAGCGGTTCAGCAGGAATGCCGAGACCGATNNNN
IIIIHIIIIIIGIIIIGGGGGGIIIIIIIIIIIIIIIFGGGIIIIDIFGFIIIIFIFFIDIIFIIHGIEHIHIDGIEHNNNN AS:124 MATCHES:48 MISMATCHES:28 GAPS:0
```
